# Dopamine supports idea originality: the role of spontaneous eye blink rate on divergent thinking

**DOI:** 10.1007/s00426-022-01658-y

**Published:** 2022-02-10

**Authors:** Sergio Agnoli, Serena Mastria, Marco Zanon, Giovanni Emanuele Corazza

**Affiliations:** 1 Department of Life Sciences, University of Trieste, Via Weiss 2, 34128 Trieste, Italy; 2 Marconi Institute for Creativity (MIC), Villa Griffone, Via dei Celestini 1, 40037 Sasso Marconi, Italy; 3 Department of Electrical, Electronic, and Information Engineering “Guglielmo Marconi”, University of Bologna, Viale del Risorgimento 2, 40136 Bologna, Italy; 4 Scuola Internazionale Superiore Di Studi Avanzati, SISSA, Trieste, Italy Via Bonomea, 265, 34136; 5 Université de Paris and Univ Gustave Eiffel, LaPEA, 92100 Boulogne-Billancourt, France

## Abstract

The neurotransmitter dopamine plays a crucial role in human creative behaviour. Specifically, striatal dopamine seems to be associated with specific dimensions of divergent thinking performance, especially with categorical diversity (flexibility) of ideas. In experimental contexts, spontaneous Eye Blink Rate (sEBR) has been used as a proxy for striatal dopamine, and an inverted U-shape relationship between sEBR and flexibility has been demonstrated, such that a medium sEBR level predicts highest flexibility levels. The present study aimed at carrying out further investigations about the relationship between sEBR and idea generation through divergent thinking, specifically focusing on the relationship between idea originality and dopamine level, since originality is a key element for creativity. We asked 80 participants, whose sEBR at rest was measured, to perform an Alternative Uses Task (AUT) to measure their divergent thinking performance. Results revealed that the relationship between sEBR and originality, as measured through subjective ratings of external raters, followed an inverted U-shape function with medium sEBR being associated with highest originality scores. Moreover, and most importantly, we demonstrated that sEBR predicted originality through the mediation of flexibility. Our results provide further insights on the possible role of dopamine on divergent thinking performance, demonstrating that an adequate dopamine level may facilitate the generation of original ideas through the exploration of diverse conceptual categories (higher flexibility).

## Introduction

Creativity is one of the most powerful human behaviours, characterizing both the progress of the society and the success of individuals in their daily life. Given its inherent complexity, the extant scientific literature converges on understanding creativity as a multifaceted phenomenon entailing an interplay of various cognitive mechanisms (e.g., Benedek & Fink, 2019). From an operational point of view, creative thinking can be conceived as the ability to produce potentially original and effective outcomes such as ideas, works, products, or services. The notion of originality refers to newness, uniqueness, and non-obviousness, whereas effectiveness indicates the value or relevance of the creative outcomes in a specific domain (Corazza, 2016; Lubart, 2001; Mumford et al., 2012; Runco & Jaeger, 2012; Sternberg & Lubart, 1996).

Neurobiology and cognitive neuroscience have started to shed light on several candidate brain regions and networks supporting creative thinking, including the prefrontal cortex (e.g., Abraham et al., 2012; Barr et al., 2015; Benedek et al., 2014; De Dreu et al., 2012; Dietrich & Kanso, 2010) and the striatum (e.g., Cools & D’Esposito, 2011; Mayseless et al., 2013; Zabelina et al., 2016), which are closely related by the neurotransmitter dopamine (Alexander et al., 2007). The dopamine (DA) system indeed exerts a pervasive, modulatory effect on both prefrontal cortex and striatum, leading to a modulation of creative thinking (e.g., Flaherty, 2005; Takeuchi et al., 2010). The impact of DA on creativity is underlined by various research lines. In fact, a body of evidence highlighted the role of specific genetic markers on creative behavior, such as those related to D2 (Reuter et al., 2006; Zhang et al., 2014) and D4 (Mayseless et al., 2013) dopamine receptors, whereas other research showed the effect of methylphenidate (a psychostimulant that enhances brain dopamine levels; Depue & Collins, 1999; Gvirts et al., 2017) or tyrosine (a biochemical precursor of dopamine; Colzato et al., 2015) on creative performance when compared to placebo treatments. There are also clinical studies comparing creative behavior in patients with specific disorders characterized by evident dopaminergic dysregulation, such as patients with Parkinson’s disease (e.g., Faust-Socher et al., 2014; Polner et al., 2015) or schizophrenia (e.g., Carson et al., 2003; Eysenck, 1993), as contrasted against the behavior of healthy controls. All together, these studies converge on the conclusion that dopaminergic modulation of the fronto-striatal network in the human brain supports creative behaviour, regulating the proper balance between two key cognitive mechanisms at the basis of creative thinking, i.e., persistence and flexibility (Boot et al., 2017; Nijstad et al., 2010; Zabelina et al., 2016).

Along with the neurophysiological exploration of the dopaminergic systems and the genetic analysis of dopaminergic receptors, another research line investigated the use of a non-invasive physiological proxy of DA functioning: the spontaneous Eye Blink Rate (sEBR). An extensive body of cognitive research suggests indeed that sEBR is a useful predictor of DA in a wide variety of contexts, in particular when it comes to explore creative behaviour (Boot et al., 2017). Specifically, resting state sEBR seems to be closely associated with central DA functioning, particularly in the striatum. Although it provides a basic and indirect subcortical measure of DA, which does not distinguish between different dopaminergic pathways and receptors systems, sEBR is viewed as a reliable and easily accessible non-invasive proxy of DA levels in humans. Therefore, quite often sEBR is preferred over more invasive or expensive DA investigations based on functional imaging techniques or genetic polymorphisms (Jongkees & Colzato, 2016).

The first direct investigation on the role of sEBR on specific indicators of creative performance comes from the study of Akbari Chermahini and Hommel (2010). The authors employed sEBR to explore the predictive role of the individual DA level on divergent (the ability to generate a number of alternative responses to open-ended problems) and convergent (the ability to generate a single correct answer to close-ended problems) modalities in creative thinking. They showed that whereas convergent thinking performance benefited more from low sEBR levels, the relationship between divergent thinking, measured through the Alternative Uses Task (AUT; Guilford, 1967), and sEBR was more complex. In particular, the divergent thinking-sEBR relation followed an inverted U-shape, suggesting that an adequate (i.e., a medium) level of sEBR would improve the AUT performance. This pattern of results was specifically found when focusing on flexibility, i.e., the ability to switch between diverse semantic categories in different responses (Akbari Chermahini & Hommel, 2010) in comparison to other indicators of AUT performance (i.e., originality, fluency, elaboration). Recently, Ueda and colleagues (2016) found that individuals with a moderate sEBR at rest showed higher fluency (i.e., a higher number of ideas) than individuals with higher or lower sEBR levels. This curvilinear relationship between sEBR and AUT performance has also been confirmed from Akbari Chermahini and Hommel (2012), who investigated more closely the association between flexibility and sEBR by means of mood induction (positive or negative). Based on the assumption that positive moods may improve creativity (see, e.g., Baas et al., 2008; Davis, 2009), and that DA may mediate this relationship, authors have demonstrated that a positive mood induction (but not a negative one) increased sEBR, and that this effect was associated with enhanced flexibility scores. Interestingly, low-sEBR individuals benefitted more in terms of flexibility from a positive mood induction than medium or high-sEBR individuals. Further support comes from a recent research showing that the degree to which people experienced positive affect when they were engaged in a divergent thinking task was positively correlated with sEBR (Rooij & Vromans, 2020). Altogether, these findings seem to suggest that sEBR predicts certain measures of divergent thinking performance through a quadratic (inverted U-shape) relationship, and that the individual differences in affect are reflected in corresponding changes of sEBR. Considering these findings, it is worth noting that whereas a medium sEBR is associated with higher flexibility and fluency levels, originality has not yet been shown to benefit from an adequate sEBR level.

### The research problem

Theoretical frameworks of creativity research have given great importance to the dimension of originality in divergent thinking, as originality is one of the defining elements of the concept of creativity (Corazza, 2016; Rothenberg & Hausman, 1976; Runco, 1988; Runco et al., 2005; Runco & Jaeger, 2012). But what does originality mean in operational terms? Originality is often evaluated in terms of quality such as *uncommonness*, *remoteness*, and *cleverness* of an idea (Guilford, 1967; Silvia et al., 2008). These quality criteria are taken into consideration in the study of divergent thinking since they have a clearly defined association with the concept of creativity (Forthmann, Bürkner, et al., 2019; Forthmann, Oyebade, et al., 2019). This quality dimension of originality is usually scored by rater-based methods, which require at least two judges with a sufficient experience in creativity research to evaluate response originality on a Likert scale (see Reiter-Palmon et al., 2019 for an overview). Beside rater-based methods, the evaluation of response originality can be determined also on the basis of quantitative criteria, i.e., using frequency-based scoring methods. Frequency-based (FB) originality measurement is based on the computation of the statistical frequencies of participants’ ideas so that uncommonness of responses within a sample of participants can be extracted. Although frequency-based scoring methods are appealing because of their objectivity for creativity measurement (see Runco, 2008), they raise important methodological problems. First, frequency-based originality score is often confounded by fluency and blinded by fuzzy responses. Second, this procedure works only when the sample size is sufficiently large (Forthmann et al., 2020). Last, the frequency estimates for scoring responses as "uncommon" inevitably involve subjective evaluations and adaptations in order to identify whether certain responses are actually the same as or different from each other (Forthmann, Bürkner, et al., 2019; Forthmann, Oyebade, et al., 2019; Reiter-Palmon et al., 2019). In conclusion, Rater-Based (RB) originality measurement is recommended instead of FB methods for smaller samples (e.g., Hass, Rivera, & Silvia, 2018; Silvia et al., 2008), and furthermore is able to capture both remoteness and cleverness indicators of originality besides uncommonness (Forthmann et al., 2017; Silvia et al., 2008), i.e., qualitative indicators of ideas beside quantitative ones.

As previously mentioned, in the exploration of the connection between divergent thinking performance and sEBR, with the exception of flexibility and fluency, the ability to generate original ideas, which is the strongest predictor of creativity (Acar et al., 2017), did not appear to benefit from an adequate dopamine level (see Akbari Chermahini & Hommel, 2010; De Rooij et al., 2020). This is quite surprising given the strong interrelationship between originality and flexibility, as the exploration of diverse conceptual categories (flexibility) consistently emerges to facilitate the generation of original ideas (e.g., Acar et al., 2019). However, all previous studies exploring the relationship between performance in divergent tasks and dopaminergic functioning measured by sEBR have mainly employed a frequency-based scoring of originality (Akbari Chermahini & Hommel, 2010, 2012; De Rooij et al., 2020; Ueda et al., 2016). Since different scoring methods correspond to different conceptualization of originality in divergent thinking, it is possible that the lack of a connection between sEBR and originality (see Akbari Chermahini & Hommel, 2010, 2012; De Rooij et al., 2020) could be due to the adopted scoring method, i.e., to the focus on quantitative indicators of ideas originality (i.e., frequency) instead of qualitative indicators of originality (i.e., remoteness, cleverness), which research highlighted as the most powerful indexes of creativity (Forthmann et a., 2019).

The present study, therefore, aimed at contributing to the clarification of the relationship between DA and divergent thinking performance, assessing whether idea originality, measured through a rater-based method, can benefit from medium dopamine levels, assessed indirectly by sEBR at rest. When considering the quality of ideas beyond the mere characterization of "uncommonness", it can be hypothesized that divergent thinking performance might benefit from an adequate level of dopamine not only when focusing on flexibility or fluency, but also when measuring originality. We, therefore, expected to find that an adequate level of sEBR, which indirectly indicates dopaminergic signal transmissions, would be associated with a greater originality of the responses as measured by external raters. Second, considering the typical positive linear relationship between flexibility and originality (Acar et al., 2019; Christensen et al., 1957), and the consistent results showing a relationship between sEBR and flexibility, it is suggested that the relation between sEBR and originality could be mediated by flexibility. The direction of this relation (i.e., flexibility as mediator of the relationship between sEBR and originality) is supported by previous studies showing that the greater use of diverse categories during ideation, which is the cognitive mechanism of flexibility (Mastria et al., 2021), tend to yield more original ideas (Acar et al., 2019; see also Christensen et al., 1957), and not the other way around. Proving our hypothesis would provide further insights into the role of sEBR as a predictor for originality and flexibility in divergent thinking, possibly suggesting that adequate levels of DA might lead to original ideas through the exploration of diverse conceptual categories (higher flexibility). To ensure compatibility with previous results (Akbari Chermahini & Hommel, 2010, 2012; De Rooij et al., 2020; Ueda et al., 2016), we asked participants to perform an alternate uses task (AUT; Guilford, 1967) as divergent thinking measure, whereas their spontaneous eye blink rate at rest was measured.

## Method

### Participants

Eighty healthy young adults took part in the study for monetary reward. Age ranged from 18 to 27 years (*M* = 20.87, SD = 2.25). Participants were recruited from diverse departments at the University of Bologna (Italy) and gave written informed consent to participate in the research. All participants had normal or corrected-to-normal vision, and none of them reported current or past neurological or psychopathological problems. Participants filled a screening questionnaire assessing their medical history, which was adapted from one that is regularly used in non-invasive brain stimulation studies (see Rossi et al., 2009, 2011). The experimental protocol conformed to the declaration of Helsinki and was approved by the Ethical Committee of the University of Bologna. Due to low EEG signal quality, eye-blink rate data were not available for 7 participants. Analyses were thus performed on a total of 73 participants. A statistical power analysis was conducted using G*Power 3.1 (Fault et al., 2009) to determine the appropriate sample size to perform the hypothesized mediation analysis. The analysis identified a sample size of 55 as a lower limit of the number of participants needed to achieve a power of 0.80 at the alpha level of 0.05 (Cohen, 1988) with three predictor variables, by assuming a medium effect size of 0.15.

### Procedure

The experiment reported in this article was part of a larger study over three sessions of neurofeedback training including EEG registration with a G.tec g.HIamp amplifier (Guger Technologies OG, Austria), the results of which were published in Agnoli et al. (2018). Participants were seated in a chair in a sound-attenuated room, signed informed consent form, and received an explanation of the whole experimental session. Participants’ sEBR was measured in a 3-min eyes-open resting state as described in the former study, specifically before performing the Alternative Uses Task, which required generating alternative uses for conventional, everyday objects. All participants were debriefed after the session.

### Spontaneous eye-blink rate

Eye-blinks were recorded for 3-min eyes-open segments under resting conditions. Participants were comfortably sitting in front of a black 19″ LCD monitor, located about 1 m from the participant. Each participant was asked to look at the computer screen in a relaxed state; nothing specific was said about blinking in resting state. Additionally, we asked participants to avoid alcohol and nicotine consumption 24 h before the recording. The blink detection was performed using a freely available MATLAB toolbox called BLINKER pipeline (Kleifges et al., 2017). The BLINKER algorithm selects the best channel among an arbitrary number of EEG channels, allowing the optimal identification of blinks. We used frontal EEG channels (Fpz, Fz, Fp1, Fp2, F3, F4, FC3, FC4), as they usually provide the best signals for capturing blinks. Before proceeding to blink detection, the sensors signal was bandpass filtered between 1 and 20 Hz. Potential blinks fall into intervals during which the signal is greater than 1.5 standard deviations above the overall signal mean, duration is longer than 50 ms, and interblink interval is at least 50 ms. These criteria allowed to exclude many small rapid eyes movements, preserving actual blinks. Among these, only blinks showing a correlation with the tent-like shape which was higher than 0.90 have been considered. As a last check, only the blinks with the positive amplitude velocity ratio (pAVR) < 3 were chosen in order to eliminate saccades. The number of blinks per minute was considered for analyses, which represents the spontaneous Eye Blink Rate.

### Alternative uses task (AUT)

The paper and pencil version of the AUT was used. Each participant was asked to think of and write down alternative uses for five everyday objects (e.g., a hat, a hammer). Specifically, the instructions to participants were to generate as many alternative uses as they could think of for each object in 2 min, for a total of 10 min. Objects were all new and randomly presented across participants. Participants generated a total of 1673 responses for the presented objects. Four measures of participants’ creative performance were derived from the AUT: flexibility, FB originality (in the form of statistical infrequency), RB originality (using external raters), and fluency.

Flexibility was calculated by averaging at the subject level the total number of different conceptual categories utilized per each object, according to pre-existing categories extracted ad hoc based on our data set (Reiter-Palmon et al., 2019).

The FB originality measure was obtained by counting the statistical frequencies of each response, compared to the total amount of responses generated for each object by all subjects, such that 1 corresponds to the lowest frequency (rare responses) and 0 corresponds to the highest frequency (common responses). To give an example, if the response *vase* appeared 8 times as a response as an alternative use of a *brick* among 20 productions generated for this object by the sample, the relative frequency of this response would be 0.40 (i.e., 8/20) and, consequently, its statistical infrequency would be 0.60 (i.e., 1–0.40; see Forthmann et al., 2020). Before calculating statistical infrequency, the database was adequately adapted in order to take into account response similarity, according to the following criteria: (i) unnecessary words such as “*used for*” have been deleted; (ii) singular and plural words have been made consistent; (iii) diminutives have been avoided; and (iv) other slight changes have been introduced to facilitate the identification of equivalent responses (see Reiter-Palmon et al., 2019).

Regarding RB originality, two expert^1^ judges were involved to independently rate the originality of each participant’s response (Silvia et al., 2008). For each object, the responses were transcribed into a spreadsheet and alphabetically ordered within each object. The ID associated with each participant was then hidden. This procedure guaranteed that the evaluation was not biased by the serial position of the responses, the total number of the responses in the set, the participant who generated the response, and the preceding and successive responses. The judges were required to read all responses before scoring them. According to one of the most accepted scoring methods (see Silvia et al., 2008), response originality was rated on a 1 (= *not at all original*) to 5 (= *highly original*) scale. Responses should be uncommon, remote, and clever to be judged as original. As suggested by past literature when an overall quality score is employed in a study based on subjective ratings, raters were instructed to integrate mentally these three dimensions before scoring each idea in the sample (Forthmann et al., 2017). Specifically, an idea was considered original when it was (i) uncommon; otherwise, an idea that everyone can think of is not original by definition; (ii) remote, which refers to the distance of an idea to what is commonly thought, and (iii) clever, which is related to the notion of imaginativeness, smartness, and funniness (Silvia et al., 2008, see also Forthmann et al., 2017). In the present study, as indicated by the procedure described in Silvia et al. (2008), these three dimensions had to be weighted by the raters and ideas were scored as highly original when they were high on all these three dimensions. Inter-rater reliability calculated on the total number of uses produced by participants was good (ICC = 0.88). In case of huge discrepancies between ratings, the judges reviewed their responses and assigned a score by consensus. The mean originality score for each idea was calculated from the ratings of the two judges and an average originality score per participant was derived as average of responses originality for the 5 objects.

Fluency was finally scored as the total number of valid responses generated by each participant.

## Results

### Correlation analyses

SPSS 26 (SPSS Inc., Chicago, USA) and R (R Core Team, 2019) were used to conduct analyses. Results of Pearson Correlation among the divergent thinking measures (see Table 1) showed that whereas RB originality was positively correlated to flexibility and negatively to fluency, FB originality failed to correlate with the remaining AUT measures. Finally, a negative relationship between flexibility and fluency was found.

**Table 1:** Correlations among divergent thinking (AUT) performance measures

	*RB Orig*	*FB Org*	*Fluency*	*Flexibility*
RB Orig	_			
FB Orig	− .030	_		
Fluency	− .240*	.135	_	
Flexibility	.567**	− .091	− .376**	_

*RB Orig.* Rater-Based Originality, *FB Orig.* Frequency-based Originality

^*^p < .05, **p < .01

### Regression analyses

**Fig. 1:**
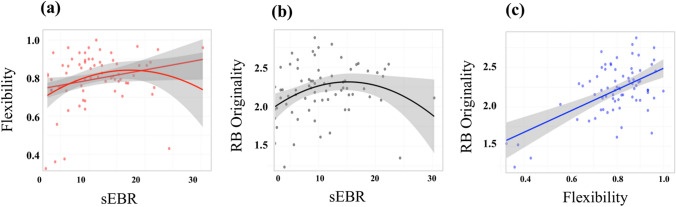
The figure depicts significant relationships between spontaneous eye blink rate (sEBR) and divergent thinking (AUT) performance measures: **a** flexibility, **b** RB originality. Panel (**c**) depicts the relationship between flexibility and RB originality. Points show individual data. The grey background indicates 95% predicted confidential intervals

Hierarchical multiple regressions models were used to examine the relationship between sEBR and each divergent thinking measure (i.e., flexibility, RB originality, FB originality, fluency) and were interpreted in terms of explained variance (*R*^*2*^) and significance of standardized regression coefficients (ß) estimates. Since flexibility, fluency, and FB originality data contain outliers (> 3 standardized residuals), robust regression models were performed, which are less sensitive to possible effects of outliers, using the *robustbase* library (Rousseeuw et al., 2009) in R. In the first step, we carried out a linear model on each divergent thinking index, using sEBR as predictor. In the second step, we tested the quadratic model, adding to the previous linear regression model the squared factor of sEBR. Results of the multiple regression analyses are shown in Table 2. As shown in Fig. 1a, sEBR reliably predicted flexibility score of the divergent thinking measure with a resulting quadratic fit (inverted U-shaped), supporting the trend found in previous findings (Akbari Chermahini & Hommel, 2010, 2012). Moreover, the linear relationship of sEBR with flexibility almost reached significance. Whereas both linear and quadratic fits of sEBR on FB originality and fluency were far from significance (*ps* > 0.17), the relationship between sEBR and RB originality (derived from the rated-based method) showed a significant quadratic inverted *U*-shaped pattern, with medium sEBR being associated with the highest originality score (see Fig. 1b). Finally, the typical linear effect of flexibility on originality emerged in our data in association with RB originality (*R*^*2*^ = 0.69, ß estimate = 1.57, *t*(71) = 4.68, *p* < 0.001, see Fig. 1c), but not when associated with FB originality (*R*^*2*^ = -0.003, ß estimate = 0.001, *t*(71) = 0.24, *p* = 0.811).

**Table 2:** Hierarchical multiple regression of the relation between sEBR and each divergent thinking (AUT) performance measure

	Alternative uses task								
	Flexibility		RB Originality			FB Originality		Fluency	
	*Step 1: Linear*	*Step 2:* *Quadratic*	*Step 1: Linear*	*Step 2: Quadratic*		*Step 1: Linear*	*Step 2: Quadratic*	*Step 1: Linear*	*Step 2: Quadratic*
**sEBR**	**.006**^**+**^ **(.003)**	.025* **(.012)**	.008 (.006)	.058** **(.021)**		.001 (.000)	− .001 (.000)	− .146 (.135)	− .919 (.572)
**sEBR**^**2**^		− .001* **(.001)**		− .002* **(.001)**			.001 (.000)		.029 (.021)
*R*^*2*^	.277	.289	.025	.105		.004	.004	.011	.040
*ΔR*^*2*^	.277	.012	.025	.079		.004	.000	.011	.028
*t* **(**sEBR)	**1.721**	**2.066**	1.344	**2.803**		.511	− .057	− 1.082	− 1.607
*t* **(**sEBR^2^)		− **2.069**		**-2.504**			.175		1.381

Linear (Step 1); Quadratic (Step 2). Numbers in the first two rows represent standardized regression coefficients estimates and standard errors (between parentheses); *sEBR* corresponds to spontaneous eye-blink rate, *RB Originality* Rater-Based Originality, *FB Originality* Frequency-based Originality

*p < .05, **p < .01, ^**+**^*p* = .089

### Mediation analyses

Given the evidence for a curvilinear effect of sEBR on originality in the hierarchical multiple regression analyses, and the typical linear effect of flexibility on RB originality, we used the Medcurve SPSS macro (Hayes & Preacher, 2010) to estimate the indirect effect of sEBR on originality through the flexibility mediator. Instantaneous indirect effects can be analyzed as Theta (θ), which indicates the strength of the indirect effect as a function of specific values (low, moderate, and high) of the predictor. Theta is calculated using the following formula: Theta = (*a*_1_ + 2_a2_X)*b* (Hayes & Preacher, 2010). Here, the terms *a*_1_ + 2 _a2_X capture path *a*, where *a*_*1*_ is the coefficient path of the linear trend, *a*_*2*_ is the coefficient path of the quadratic trend, and *X* is the predictor; since the indirect effect is calculated via multiplication of path *a* and path *b*, the term in brackets has to be multiplied with the coefficient of the linear trend of path *b*. Specifically, Theta explains the rate at which a variation in the predictor (*x*) changes the criterion (*y*) indirectly through changes in the mediator (*m*). A resampling bootstrap technique provides Confidence Intervals (CI) for indirect effects.

Since sEBR and flexibility emerged to be related both in a linear and in a quadratic modality (even if the linear relation was only marginally significant), we tested these two modalities in two distinct models. First, we specified the *X* (i.e., sEBR) → M (i.e., flexibility) and M → Y (i.e., originality) paths as linear and the *X* → Y path as quadratic, and used 5000 bootstraps to estimate the 95% CI for the test parameter *θ*. Results revealed that, when relating flexibility to sEBR in a linear way, the indirect effect of sEBR on originality was not significant (*θ*_*x* = 4.71, 10.76, 16.81_ = 0.006, 95% CI − 0.010 to 0.016), showing that the linear relationship between sEBR and flexibility is not a mediator of the curvilinear relation between sEBR and originality.

**Fig. 2:**
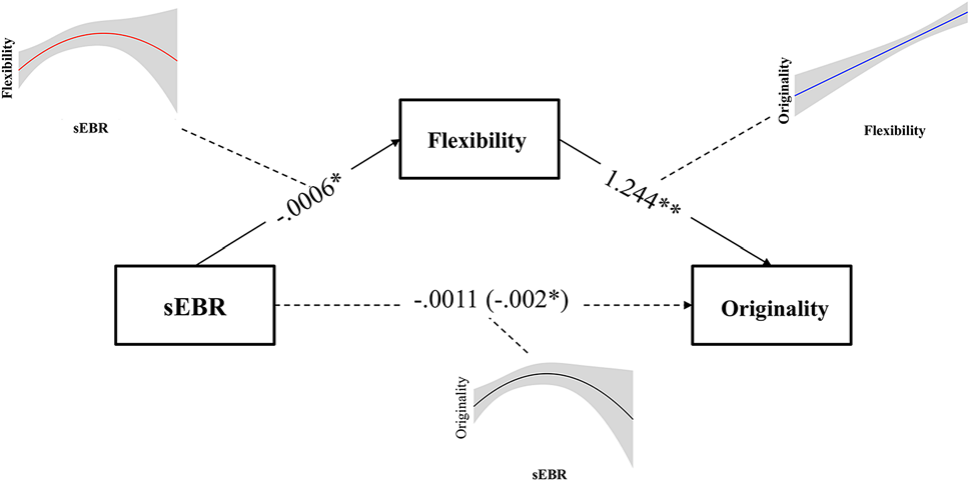
Mediation analyses representing the indirect effect of spontaneous eye blink rate (sEBR) on originality via the flexibility mediator. Unstandardized coefficients are reported in the picture; *p < .05, **p < .01

In a subsequent mediation analyses, we defined the *X* → *M* and the *X* → *Y* paths as quadratic and *M* → *Y* path as linear, using the same 5000 bootstraps to estimate the 95% CI for the test parameter *θ*. Interestingly, after the inclusion of the quadratic relationship between sEBR and flexibility, the indirect effect of sEBR on originality was significant, indicating that the curvilinear relationship between sEBR and flexibility was a significant mediator of the curvilinear effect of sEBR on originality on low (*θ*_x = 4.71_ = 0.0192, 95% CI 0.002 to 0.049) and medium (*θ*_x = 10.76_ = 0.0100, 95% CI 0.002 to 0.021) values of the sEBR; however, flexibility was not a significant mediator on high values of sEBR (*θ*_x = 16.81_ = 0.000, 95% CI − 0.022 to 0.010). For low and medium values of sEBR, the indirect effect of sEBR on originality was thus significant, suggesting that positive differences in sEBR were associated with positive differences in originality through the mediation of flexibility, whereas at high sEBR levels this mediated effect disappeared. A mediational diagram with unstandardized coefficients is depicted in Fig. 2.

## Discussion

The main aim of the present study was to investigate whether idea originality can benefit from an adequate sEBR level at rest, possibly clarifying the connection between divergent thinking performance and DA level. As stated by Runco et al. (2011), whereas other indexes used to measure creative production (such as fluency and flexibility) are meaningful for the understanding of the dynamics in the generation of creative ideas through divergent thinking, originality is the key to creativity. We, therefore, explored response originality using different scoring methods and specifically a frequency-based method, essentially defining the uncommonness of the responses within the sample (FB originality), and a rater-based method (RB originality), which included also qualitative elements in the scoring of response originality. Confirming previous literature (Forthmann et al., 2017; Forthmann, Bürkner, et al., 2019; Forthmann, Oyebade, et al., 2019), the correlational analyses showed that these two methods provide distinct originality measures. Thus, even if they share a common criterion in the scoring of originality (uncommonness), the qualitative dimensions (remoteness and cleverness) that are additionally considered in the RB originality do allow to provide a different measurement for originality. In fact, the expert judges involved in the present study were instructed to weigh both quantitative and qualitative dimensions in their evaluations. As hypothesized, individual sEBR at rest was able to predict the originality of ideas through a non-linear inverted U-shaped relationship, such that originality benefitted most from medium sEBR. This result specifically emerged when the qualitative analysis of response originality was taken into account.

To the best of our knowledge, the present study addresses for the first time originality scored by trained expert raters when exploring the relationship between divergent thinking performance and sEBR. We demonstrated that, when evaluating responses in terms of quality, changes in sEBR significantly predicted the increase of originality, resulting in a quadratic fit. Taking sEBR as a proxy of the individual level of dopaminergic functioning, this suggests that originality, codified in terms of uncommonness, remoteness, and cleverness, could be related to DA via a curvilinear relationship. This finding is in line with the assumption that original responses are not merely uncommon, but also remote and clever (Forthmann et al., 2017; Silvia et al., 2008), and it demonstrates that the consideration of the interplay of these qualitative indicators make a difference in the study of the relationship between divergent thinking and DA. In fact, all previous studies exploring the relationship between originality, intended only as uncommonness, and DA measured through sEBR did not find any relationship between these two variables. We suggest that this lack of association might be related to a methodological gap, since past studies have mainly used quantitative indicators of idea originality (Akbari Chermahini & Hommel, 2010, 2012; De Rooij et al., 2020; Ueda et al., 2016). Consistently, the results of past research are confirmed also in the present study, where no association emerged between sEBR and originality, when it was scored only on the basis of its uncommonness dimension, using the quantitative index of FB originality. These results are in line with past research on divergent thinking that showed that the subjective ratings based on uncommonness, remoteness, and cleverness are clear indicators of the overall quality dimension of an idea (see Wilson et al., 1953), which is also most closely related to the concept of creativity, rather than quantity (Runco, 2011).

When it comes to the other indexes of divergent thinking, we consistently confirmed the results of past research. We indeed found a quadratic, inverted U-function between the resting state EBR and AUT flexibility; moreover, also a linear relationship between these measures was (marginally) observed. This result confirmed previous findings showing that sEBR is able to predict flexibility in divergent thinking (Akbari Chermahini & Hommel, 2010, 2012), which here conceptually reflects the ability to break the set of typical associations and to consider different alternatives during idea generation (Nijstad et al., 2010). Closely related to this result, a number of studies reliably suggest that creative cognition benefits from flexible processing, and that dopaminergic striatal modulation regulates this process (Boot et al., 2017). Flexibility is indeed considered one of the key cognitive mechanism supporting creativity (Nijstad et al., 2010). Our results additionally showed that sEBR was not able to predict fluency in the divergent thinking production. This confirms previous findings on the relationship between sEBR and fluency (Akbari Chermahini & Hommel, 2010, 2012), even if there is a lack of consensus in literature on the strength of this association, with some research showing a significant curvilinear relationship between these two variables (Ueda et al., 2016, who, however, used relatively short response time windows in the AUT task, leading to high correlations between fluency and flexibility). Finally, confirming an abundant amount of research on divergent thinking (Acar et al., 2019; Christensen et al., 1957; Nijstad et al., 2010), we confirmed that flexibility and originality were linearly related, so that the ability to flexibly produce alternative responses was predictive of higher originality levels.

Based on the reliable relationship existing between sEBR and flexibility (Akbari Chermahini & Hommel, 2010, 2012), which we consistently confirmed in our data, and on the fact that flexibility was beneficial to originality (e.g., Nijstad et al., 2010), the second aim of our study was to test whether the relationship between sEBR and originality would be mediated by flexibility, possibly providing further insights into the role of DA on originality and flexibility in divergent thinking. As hypothesized, results revealed that flexibility, quadratically related to sEBR, was a significant mediator of the curvilinear relationship between sEBR and originality, which was linearly related to flexibility. Put simply, our findings seem to suggest that adequate (medium) sEBR levels non-linearly predict the originality of divergent thinking responses through the exploration of diverse conceptual categories (higher flexibility). These results rise a series of interesting theoretical and empirical implications.

First, our results are consistent with the claim that originality in divergent thinking tasks is a multifaceted concept than entails not only the uncommonness dimension but also the remoteness and cleverness indicators (Forthmann et al., 2017; Silvia et al., 2008). In fact, it makes sense to assume that, similarly to the flexibility indicator of divergent thinking performance (Akbari Chermahini & Hommel, 2010, 2012), originality, when it is measured taking into account its quality dimension—which is the most representative indicator of creativity (Forthmann et a., Forthmann, Bürkner, et al., 2019b)—is driven by dopamine. Second, our findings revealed that originality is related to resting state sEBR in a quadratic way, showing that this main requisite for creativity, along with flexibility, benefitted most from medium sEBR. This observation provides strong support for contemporary scientific evidence relating creativity to DA (Murphy et al., 2013; Takeuchi et al., 2010; Zabelina et al., 2016; see Boot et al., 2017 for a review). Indeed, differently from low dopaminergic functioning, which can partially determine lack of motivation, positive mood and desire, or from high dopaminergic functioning, which otherwise can lead to elation or mania, a relatively medium (or adequate) dopamine level might allow a better divergent thinking performance measured in terms of idea quality. Lastly and most importantly, our data demonstrate that the role played by sEBR at rest in the ability to produce original idea is quite complex. On the one hand, flexibility is linearly related to originality, since during the course of divergent thinking ideas tend to become more original because it is more probable that they are drawn from new or more remote conceptual categories, (Acar et al., 2019), on the other hand, sEBR is related to flexibility via a curvilinear relationship. It follows, as shown by our results, that the quadratic relationship between sEBR and originality can be explained as a function of the relationship between sEBR and flexibility. Despite its complexity, the frame is clear: an adequate amount of spontaneous dopaminergic activation, as measured by sEBR, may facilitate the generation of original ideas through the influence exerted on the ability to switch between different categories. As a consequence, an optimal level of dopamine would be able to push divergent thinking towards the switching between more differentiated and remote associations, thus potentially leading to more original ideas, as measured subjectively by external raters.

### Limitation and future directions

It is important to underline that sEBR, as a measure of DA level, provides only an indirect, subcortical measure of dopaminergic functioning, which for instance does not allow distinguishing between different dopaminergic pathways or receptors systems. Future work will have to provide more accurate and detailed predictions with regard to the relationship between dopamine and ideas originality using direct DA measurements. Related to this topic, considering that dopamine is typically associated with positive mood, in the context of the findings emerging from the present study, it would be interesting to introduce a manipulation of participants’ emotional states in order to explore the effect of sEBR changes as reflections of phasic dopaminergic changes on divergent thinking originality and flexibility.

At the methodological level, even though mediation analysis investigates prediction mechanisms and involves causal inference by definition, the data used here to test mediation analysis can be viewed as correlational. It is, therefore, worth highlighting that our data are limited in their capacity to yield clear conclusions regarding causality. Moreover and finally, although we did not find, consistently with previous studies, a relationship between sEBR and FB originality, it cannot be excluded that this relationship could emerge in the case of the involvement of a larger sample of participants, since recent research highlighted that the measurement precision of frequency-based originality scoring can be strongly increased through the use of large sample sizes (Forthmann et al., 2020).

## Conclusions

To summarize, the present study offers a novel evidence on the mechanisms underlying the role played by spontaneous eye blinks rate on divergent thinking performance. We first demonstrated that greater originality, measured in terms of quality, was associated with medium sEBR levels in comparison to lower or higher sEBR levels. And, second, we found that this quadratic, inverted U-shaped relationship between sEBR and originality is mediated by flexibility. All together, this research sheds new light on the functional relationship between dopamine and creative-divergent behavior.

## Footnotes

1 Within the consensual assessment technique for creativity (Amabile, 1982), "expert" is defined as any individual familiar with the domain in question. In our study the two raters could be considered experts for the present purposes, since they had expertise in scoring AUT responses. Specifically, the raters were highly trained in the evaluation of originality in divergent thinking tests, showing in the present study a good consistency and agreement between each other (ICC = 0.88).
